# Novel targets of miR-30, a microRNA required for biliary development

**DOI:** 10.12688/f1000research.2-197.v1

**Published:** 2013-09-24

**Authors:** Claire L. Le Guen, Joshua R. Friedman, Nicholas J. Hand

**Affiliations:** 1 Division of Gastroenterology, Hepatology, and Nutrition, The Children's Hospital of Philadelphia, Department of Pediatrics, Perelman School of Medicine, University of Pennsylvania, Philadelphia, PA 19104, USA

## Abstract

MicroRNAs have been found to play a profound role in embryonic and post-natal development through their regulation of processes such as cell proliferation, differentiation, and morphogenesis. The microRNA-30 (miR-30) family is necessary for vertebrate hepatobiliary development; however, the mechanism through which miR-30 regulates these processes is not fully understood. Here, we identify genes directly regulated by miR-30 that have been characterized as key developmental factors. The targets were confirmed via a luciferase reporter assay, following exogenous over-expression of miR-30a and miR-30c2 in cultured cells. Five novel miR-30ac2 targets were identified using this approach, all of which play crucial roles in hepatobiliary development or are involved in hepatocellular carcinoma and cholangiocarcinoma.

## Background

MicroRNAs are short non-coding RNAs that regulate cell function, differentiation, organ development, and disease states (reviewed in ^1– 4^). MiRNAs regulate gene expression via the RNA-induced silencing complex (RISC), leading to translational repression and/or transcript degradation ^5^. The identification of gene targets for individual miRNAs is key to understanding their function, thus necessitating accurate methods to identify significant mRNA targets. The prevailing methods of miRNA target identification begin with computational algorithms based on free energy change calculations of complementary binding, other sequence features, and evolutionary conservation ^6, 7^. However, false positive rates are high, as interactions may be predicted in physiological settings in which targeting does not naturally occur ^8^. The data gathered from these computational methods are typically validated by luciferase reporter assays in cultured cells, in which the activity of the relevant miRNA is altered (i.e., by miRNA over-expression or repression).

MiR-30a, expressed as part of an intronic cluster with miR-30c-2, is required for hepatobiliary development in zebrafish ^9^. In our previous study ^9^, we found that within the fetal mouse liver and infant human liver, members of the miR-30 family are predominantly expressed within the biliary primordium, post-natal bile ducts, and hepatocytes. Using a zebrafish model of miR-30a deficiency, we confirmed that miR-30a plays a critical role in vertebrate bile duct development ^9^. The next step in understanding the function of the miR-30 family in biliary development is to identify the genes that it targets.

Using gene expression profiling in cultured hepatoblasts ^9^, we previously identified a set of mRNAs whose levels are increased following antisense oligonucleotide (ASO)-mediated inhibition of miR30a ^9^. From this dataset, we have selected 7 novel candidate targets ( *Ccne2*, *Celsr3*, *Mdm2*, *Mtdh*, *Smad1*, *Twf*, and *Timp3*) on the basis of biological relevance and two bioinformatic prediction methods: Targetscan and PITA ^6, 7^. In addition, we evaluated four candidate targets previously tested via ASO-mediated miR-30a inhibition in cultured cells ( *Ak1*, *Tnrc6a*, *Egfr*, and *Inhba*) ^9^ ( Table 1). All 11 candidates were tested using luciferase reporter assays coupled with over-expression of miR-30a. Here we show that several of these candidate target genes - selected on the basis of their known roles in growth and morphogenesis - are direct miR-30a targets.

**Table 1.** Candidate miR-30 target genes.

Gene	Function
Ak1	Adenylate kinase
Ccne2	Cell cycle control
Celsr3	Planar cell polarity
Inhba	BMP/TGF receptor signal transduction
Mdm2	Regulation of p53
Mtdh	Epithelial-to-mesenchymal transition; tumor metastasis
Smad1	BMP/TGF receptor signal transduction
Timp3	Matrix metalloproteinase
Tnrc6a	RISC component
Twf	Control of actin turnover

See text for references.

## Methods

### Plasmid construction

The 3’ untranslated regions (UTRs) of predicted target genes were amplified from C57B/6 mouse genomic DNA by nested PCR and cloned into pMirCheck2 plasmids ^10^, a modified derivative of pSiCheck™-2 (Promega, Madison, WI) ( Data File 1).

The miR-30ac2 expression plasmid was subcloned into the pSLIK lentiviral vector platform ^11^ as follows: the murine miR-30ac2 cluster was amplified via nested PCR from C57B/6 genomic DNA and cloned into the ScaI, MfeI sites of the pSLIK tet-inducible, GFP-expressing entry vector pEN-TTGmiRc2. This entry vector was recombined into the pSLIK vector using Gateway® LR Clonase® II Enzyme mix (Life Technologies, Grand Island, NY).

### Mice

One male C57B/6 mouse was used as the source of PCR template DNA in plasmid construction. It was kept in standard caging in a controlled environment (12 hour/12 hour light/dark cycle; temperature 22°±2°C, in a cage with clear plastic walls (17 cm x 19 cm base, 16 cm high). It was fed standard mouse chow and water ad libitum. It was euthanized at 12 weeks of age by anesthesia with ketamine (200 mg/kg) and xylazine (15 mg/kg) (Sigma-Aldrich) followed by decapitation. All procedures were in accordance with federal and institutional guidelines under the supervision of the Children’s Hospital of Philadelphia Institutional Animal Care and Use Committee.

### Cell culture

The human embryonic kidney cell line HEK293FT (Life Technologies, Carlsbad, CA) was maintained at 37ºC and 5% CO _2_ in Dulbecco’s modified Eagle’s Minimum Essential Medium High Glucose GlutaMAX™ (Life Technologies) supplemented with 10% Tet-system approved fetal bovine serum (Clontech, Mountain View, CA). For luciferase reporter experiments, 5×10 ^4^ HEK293FT cells were seeded into 24-well plates. After 24 hours, cells were transfected with 900ng expression plasmid and 100ng of reporter plasmid per well using FuGENE® HD transfection reagent (Promega, Madison, WI). Twenty-four hours post-transfection, cells were induced with 1μg/ml doxycycline (Sigma-Aldrich, St. Louis, MO) for an additional 24 hours of outgrowth. For the over-expression validation experiment, 6×10 ^4^ HEK293FT cells transfected with 1μg of miR-30ac2 over-expression plasmid, using 3μl of FuGENE® transfection reagent (Promega). After 16 hours of outgrowth, cells were induced with 1μg/ml doxycycline and outgrown for an additional 24 hours. Total RNA was isolated using the miRVana™ miRNA Isolation Kit (Life Technologies) according to manufacturer’s instructions.

### Dual luciferase assay

Cells were washed with 1×PBS and lysed in 150μl 1×Passive Lysis Buffer (Promega). Firefly and Renilla luciferase activities were measured using the Dual-Luciferase® Reporter Assay System (Promega) on a GloMax Multi luminometer (Promega) according to manufacturer’s instructions. Relative light units were calculated as the ratio of Renilla to firefly luciferase activity, and the reporters were normalized to the control expression plasmid and to empty pMirCheck2 reporters to correct for nonspecific effects. Three biological replicates were performed for each condition.

### Real time quantitative PCR

Mature miRNAs were reverse transcribed from 10ng total RNA using the TaqMan® MicroRNA Reverse Transcription Kit and TaqMan® MicroRNA Assays (Life Technologies). qRT-PCR was performed in duplicate for 4 biological replicates. Relative expression was calculated using comparative C _T_ method and normalized to RNU44.

### Statistical analysis

Statistical significance was determined between groups using an unpaired Student’s t-test in Microsoft Excel, using a p-value ≤ 0.05 to determine significance.

## Results

### Over-expression of miR-30a and miR-30c2 in cell culture

**Figure 1.**
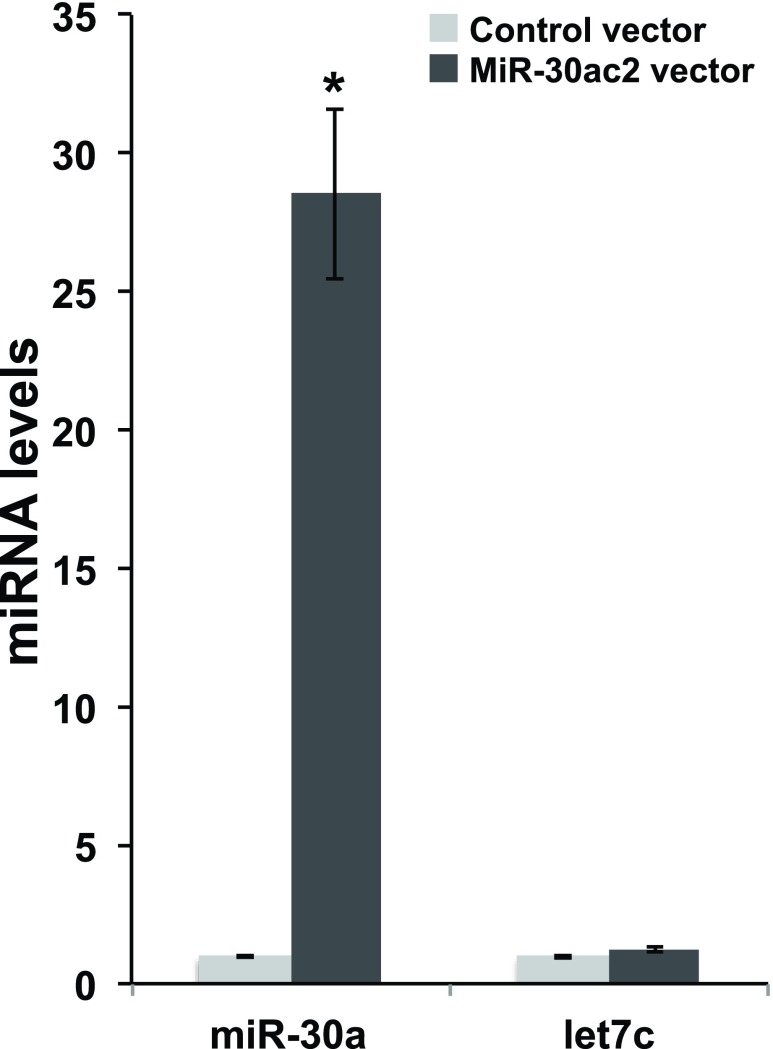
Over-expression of miR-30ac2 in HEK293FT cells. A plasmid encoding miR-30a cluster miRNAs (miR-30a and miR-30c2) or a scrambled miRNA sequence was transfected into HEK293T cells. The resulting levels of miR-30a and let-7a were measured by qRT-PCR. Error bars indicate the standard error of the mean. * p < 0.05. The data represent three separate transfections, each with three technical replicate wells.

To determine the regulatory effect of the miR-30a and miR-30c cluster (“mir-30ac2”) on the candidate target genes, we induced the over-expression of mir-30ac2 in cultured cells. We transfected a human embryonic kidney cell line transformed with the SV40 large T antigen (HEK293FT) with a doxycycline-inducible green fluorescent protein (GFP) and miR-30ac2 expression plasmid. We observed GFP-positive cells 24 hours post-induction. Mir-30a transcript levels were quantified using qRT-PCR, and revealed a 28-fold increase of miR-30a in cells transfected with the miR-30ac2 expression plasmid versus a control encoding a scrambled sequence ( Figure 1). The unrelated control miRNA let-7c was also assayed and expression levels were not significantly altered between the two treatment groups, confirming that miR-30ac2 over-expression did not have an effect on miRNA production in general.

### Confirmation of candidate miR-30 target genes

**Figure 2.**
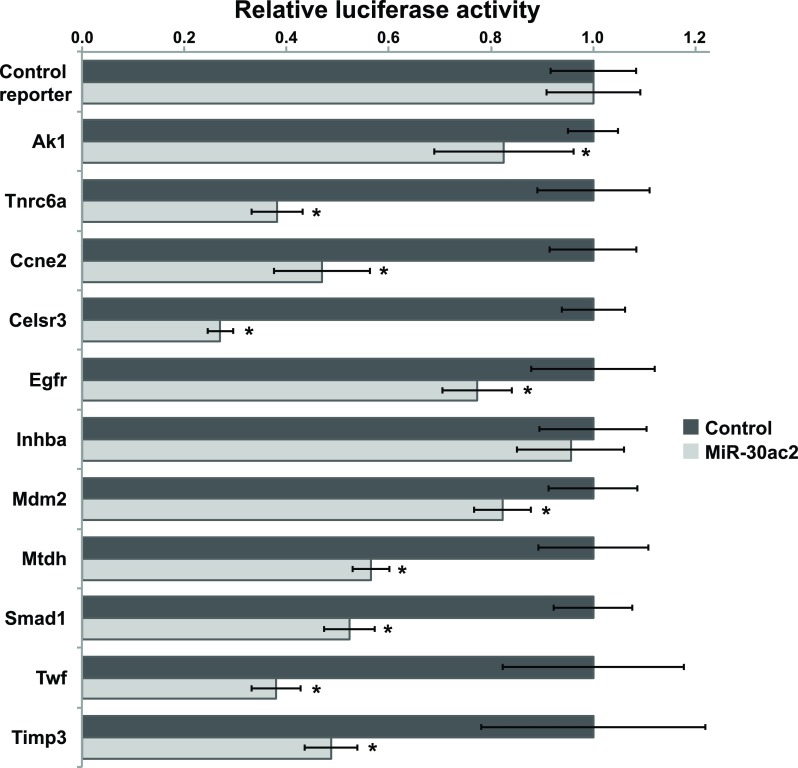
Confirmation of miR-30ac2 target genes. HEK293T cells we transfected with the miR-30ac2 expression plasmid and the indicated reporter plasmids. The ratio of firefly to Renilla luciferase activity for each reporter was normalized to that of an empty reporter. Error bars indicate the standard error of the mean. * p < 0.05. The data represent three separate transfections, each with three technical replicate wells.

We selected nine potential miR-30 target genes for further investigation and included two confirmed miR-30 target genes in our study to serve as positive controls. We constructed luciferase reporter plasmids for each of the genes of interest by subcloning each 3′-UTR immediately downstream of the Renilla (hRluc) luciferase cDNA ( Data File 1). Following co-transfection of HEK293FT cells with the miR-30ac2 over-expression plasmid and each reporter plasmid, we observed that reporters containing the 3′-UTRs of all the candidate genes were significantly down-regulated relative to the control reporter, with the exception of *Inhba* ( Figure 2). The degree of down-regulation ranged from 28–88% of basal activity.

### MiRNA target prediction

**Figure 3.**
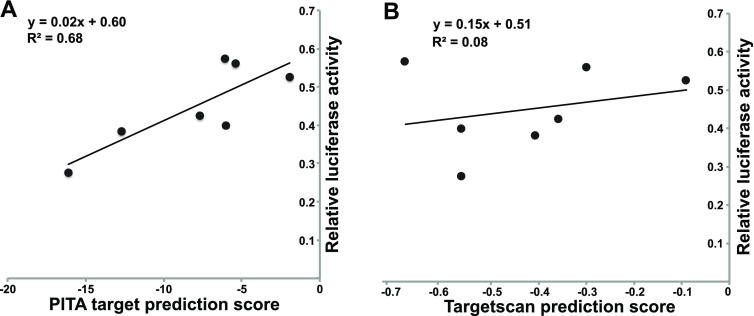
Correlation of PITA and Targetscan prediction scores with repression by miR-30ac2. The PITA (left) and Targetscan (right) scores are plotted on the x-axis, with the relative level of luciferase activity following miR-30ac2 over-expression on the y-axis.

To determine if the degree of down-regulation correlated to predicted miRNA target affinity, we correlated their PITA and TargetScan prediction scores with the reporter assay results. A slight positive correlation (R ^2^ = 0.68) was observed between the PITA target score and the reporter assay ( Figure 3). No correlation was observed between the Targetscan score and the degree of repression.

miR-30 Data for Figures Data File 1. Plasmid Sequences
Figure 1 data. Cells were transfected with an miR-30ac2 expression plasmid (“G30ac2”) or a control plasmid (“Gscr”). RNA was isolated and qRT-PCR was performed to measure the indicated transcripts. Expression was quantified using the delta-delta method.
Figure 2 data. Cells were transfected with the miR-30ac2 expression plasmid “pSLIKG-miR30ac2”) or a control plasmid (“pSLIKG-Scr”), plus the indicated luciferase reported plasmid. Renilla and firefly luciferase activities were measured in cell exctracts. The firefly luciferase activity was used to normalize for transfection efficiency, and further correction was performed to control for the effects of the reporter plasmids and the miRNA expression plasmid. Comparison was made for each reporter plasmid between the miR-30ac2 expression plasmid and the control plasmid, and averages, standard error, and p-values were calculated.
Figure 3 data. Table of PITA and Targetscan scores (if available) for the indicated UTRs, with the corresponding luciferase expression data from Figure 2.

## Discussion

The regulation of gene expression plays a critical role in establishing the complex protein patterning that is a hallmark of development and growth. While transcriptional control determines which genes have the potential to be expressed, translational control presents an additional mechanism to fine tune protein level output spatially and temporally. In the fetal liver, miR-30a and miR-30c2 are expressed in ductal plate cholangiocytes and decrease dramatically in the postnatal bile duct ^9^.

To investigate what role differential miR-30ac2 expression may be having on biliary development, we wanted to determine if miR-30ac2 directly targeted the 3′-UTR of genes that have an important function in cell growth and morphogenesis. The panel of candidate miR-30 target genes selected for validation was based on the results of gene expression profiling by microarray following miR-30a knockdown in miR-30a ASO-treated mouse embryonic liver (BMEL) cells ^9^. From this data, nine genes were selected for further validation due to the potential role they play in hepato-biliary development. Of the nine experimental targets, eight were identified as direct miR-30ac2 targets, with three of these eight targets ( *Mtdh* ^12^, *Smad1* ^13^, and *Twf* ^14^) validated as direct miR-30ac2 targets in separate studies during the preparation of this manuscript.

The novel genes identified as directly regulated by miR-30ac2 are *Ccne2*, *Celsr3*, *Egfr*, *Mdm2*, and *Timp3*. Additionally, in our study, *Mtdh*, *Smad1*, and *Twf*, were identified as miR-30ac2 targets, corroborating the results of the previous studies and validating our method. *Inhba* was the only gene included in our study that was not identified as a direct target. The targeting of these genes by miR-30ac2 support the hypothesis that the miR-30 family is required for biliary development, as the targeted genes all also have roles in hepatocyte proliferation. CcnE2 (Cyclin E2) plays a role in promoting S-phase entry from quiescence, and has also been implicated in regulatory pathways promoting liver regeneration and fibrogenesis ^15, 16^. Celsr3 (cadherin, EGF LAG seven-pass G-type receptor 3) is a cadherin important for the maintenance of planar cell polarity ^17^, an essential feature of hepatic epithelial cells ^18^. Egfr (Epidermal growth factor receptor) is an essential activator of liver regeneration ^19^, and its over-expression has also been associated with cholangiocarcoma ^20^. Mdm2 (E3 ubiquitin protein ligase) regulates tumor suppressor proteins, such as p53, by marking them for proteasomal degradation, and its aberrant expression is linked to hepatocellular carcinoma, indicating a role in liver cell proliferation ^21^. Timp3 (tissue inhibitor of metallopeptidase 3) inhibits matrix metalloproteinases which function to degrade extracellular matrix, and has also been implicated in hepatocellular carcinoma ^22^.

During the preparation of this manuscript, three of our confirmed miR-30ac2 targets were independently verified as miR-30 targets and described in other publications. Mtdh ^12^, Smad1 ^13^, and Twf ^14^. Tnrc6a and Ak1 were included in this study as verified positive controls and have been previously described ^9^. Inhba (Inhibin, beta A) was not targeted by miR-30ac2 in our assay suggesting that it is not a direct target, but does not rule out the possibility of indirect regulation.

The degree of target repression as a result of miR-30ac2 over-expression is loosely correlated to the predicted PITA score which calculates aggregate ∆∆G of all 3′-UTR binding sites. Those targets with the greatest PITA score demonstrated a high level of repression, in the order of 28–42% expression relative to our controls. The Targetscan scores did not correlate with the reporter assay results. This difference between the two methods most likely reflects their distinct algorithms; PITA relies on thermodynamic prediction of miRNA:mRNA binding affinity, whereas Targetscan places more emphasis on evolutionary conservation ^6, 7^. Overall, our results highlight the necessity of confirming predicted miRNA target genes by experimental approaches.

In conclusion, this study presents five new novel targets of miR-30a and miR-30c, all of which play roles in biliary and liver development, and additionally have implications in cancers of the liver. Given the crucial role of miR-30 in biliary development, we speculate that aberrant expression of miR-30 may be a factor in the development of human biliary diseases, and can therefore be considered as a potential target for therapeutic manipulation.
