# Supplementary material for: Novel targets of miR-30, a microRNA required for biliary development
Source: F1000Res. 2013 Sep 24;2:197. [Version 1] doi: 10.12688/f1000research.2-197.v1 (PMC3799553; doi:10.12688/f1000research.2-197.v1)
Supplement: Peer review history

# Peer review: Referee response for version 1

**Published:** 2013-10-07

**Authors:** Kalpana Ghoshal

**Affiliations:** 1 College of Medicine, Ohio State University, Columbus, OH, USA

Five different miR-30 variants (a-e), all sharing the same seed sequence, are expressed in mammals. Previously, Hand *et al. *have shown that miR-30 family members are necessary for development of the hepatobiliary track in vertebrates [ PMID: 19185580]. However, the underlying molecular mechanism through which miR-30 regulates bile duct development has not been completely understood.

In this manuscript Le Guen *et al.* report identification and validation of novel targets of two biliary-specific hepatic miRNAs, namely miR-30a and miR-30c2. To do this, authors have cloned 3’-UTRs of candidate targets into psiCHEK2 vector downstream of renilla luciferase coding sequence, and measured the ratio of renilla to firefly (internal control) luciferase activities in H293T cells co-transfected with the recombinant psiCHECK2 and an expression vector harboring miR-30ac2 gene. Using this approach, they have identified and validated 5 novel miR-30ac2 targets, namely Ccne2, Celsr3, Egfr, Mdm2, and Timp3, that are known to play an important role in hepatobiliary development, or are involved in hepatocarcinogenesis and cholangiocarcinoma development. Additionally, they confirmed Mtdh, Smad1, and Twf as miR-30ac2 targets, reported recently by others. The extent of target repression by ectopic miR-30ac2 varied among these genes and is correlated, albeit loosely, to the predicted PITA score but not the TargetScan score.

 

Overall, this is a very well written manuscript and the data is well presented. The targets of miR-30ac2, two bile duct enriched miRNA, identified in this study could explain the key function of these two miRs in liver development, and their potential causal role in hepato- and cholangio-carcinogenesis.

I have read this submission. I believe that I have an appropriate level of expertise to confirm that it is of an acceptable scientific standard.

# Peer review: Referee response for version 1

**Published:** 2013-10-01

**Authors:** Tushar Patel

**Affiliations:** 1 Transplantation Department, Mayo Clinic, Jacksonville, FL, USA

The article reports the identification and subsequent validation of several targets of miR-30a/mir-30c cluster using luciferase-based assays in a human embryonic kidney cell line. The use of computational algorithms to identify downstream targets may not always identify targets with biological significance. Thus, studies such as these are necessary to identify relevant downstream miRNA targets. The studies are well described, with adequate detail, and the conclusions are justified.  

Future studies to explore these targets could consider analyses using gene-specific 3-UTR luciferase constructs in which putative miRNA binding sites have been mutated, as well as an evaluation of their effects in the specific target cells of interest, such as hepatoblasts.

I have read this submission. I believe that I have an appropriate level of expertise to confirm that it is of an acceptable scientific standard.
